# Thoracic splenosis initially misdiagnosed as lung neoplasms: An unusual case and literature review

**DOI:** 10.1097/MD.0000000000047056

**Published:** 2026-01-02

**Authors:** Baoxiang Pei, Zhiliang Hu, Fen Pan

**Affiliations:** a Department of Thoracic Surgery, Jining First People’s Hospital, Institute of Thoracic Tumor Research, Jining, Shandong Province, China.

**Keywords:** biopsy, misdiagnosis, post-traumatic, thoracic splenosis

## Abstract

**Objective:**

Thoracic splenosis, implantation of autologous splenic tissue into the chest after diaphragmatic splenic injury, is rare and frequently mistaken for malignancy, leading to inappropriate treatment. This case highlights the risk of misdiagnosing post-traumatic thoracic splenosis as lung cancer.

**Case Report:**

A 69-year-old smoking male patient presented with 1 month of thoracic pain; computed tomography revealed a 3.3 cm left lower-lobe mass suspected as lung cancer. For the past 20 years, he underwent splenectomy after splenic and diaphragmatic injury following a severe motor vehicle accident. Normal tumor markers and history of splenic trauma raised suspicion. Computed tomography-guided biopsy showing splenic red and white pulp (Immunohistochemistry: CK⁻, Vimentin⁺) confirmed thoracic splenosis and averted needless surgery. Pain was attributed to severe coronary disease. The mass remained unchanged at 21-month follow-up and no infective complications have occurred.

**Conclusions:**

In patients with prior splenic trauma, recognizing thoracic splenosis before invasive procedures can secure the diagnosis and avert unnecessary surgery.

## 1. Background

Thoracic splenosis refers to the ectopic implantation of splenic tissue within the thoracic cavity, usually occurring after splenic rupture caused by thoracoabdominal trauma or surgery. Most cases are asymptomatic and discovered incidentally on imaging years later, often misdiagnosed as malignancies. Studies indicate that splenic autotransplantation can occur in various anatomical sites, including the abdomen, pelvis, and even the thoracic cavity. This phenomenon typically arises after splenic trauma or resection. The clinical manifestations of thoracic splenosis are nonspecific, contributing to frequent misdiagnosis. Patients may present with chest pain, dyspnea, or imaging findings of thoracic masses, which can mimic tumors or other pathologies. Asymptomatic patients generally require no treatment and may be managed with regular follow-up. Surgical intervention is reserved for cases with complications (e.g., pleural effusion, hemoptysis) or when malignancy cannot be excluded.^[1,2]^ Thus, clinicians must remain vigilant in patients with a history of splenic injury or surgery to ensure timely recognition and management of thoracic splenosis.

## 2. Case report

**Figure 1.**
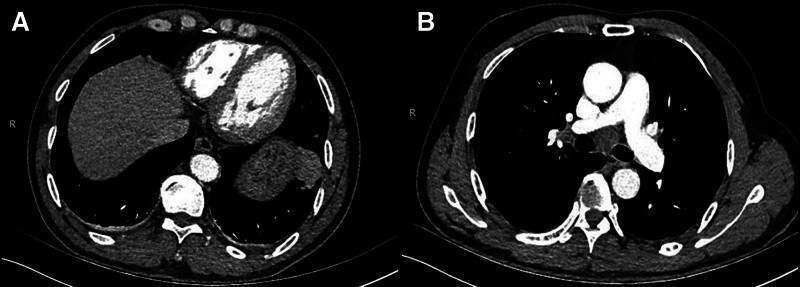
Enhanced chest CT showed that the left lower lobe pulmonary mass was approximately 3.3 cm × 2.3 cm in size, not clearly demarcated from the adjacent diaphragm (A), with slightly lobed edges and contrast-enhanced masses, enlargement of hilar or mediastinal lymph nodes (B).

**Figure 2.**
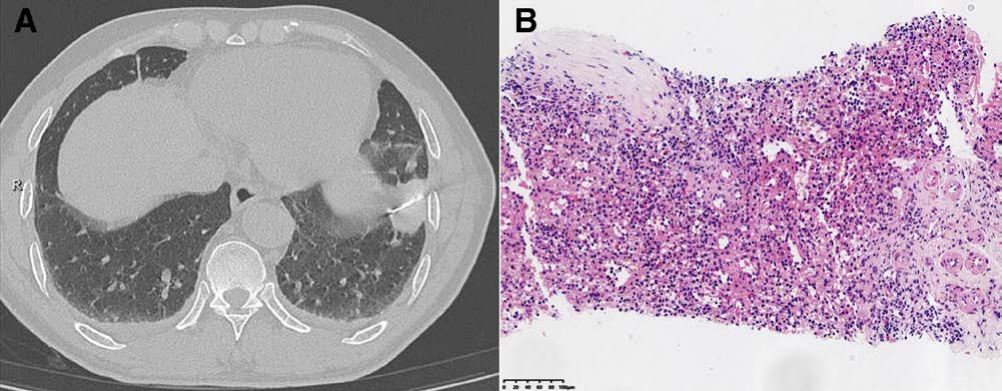
(A) CT-guided percutaneous needle biopsy along the pleura. (B) Biopsy specimen was ultimately confirmed splenic tissue.

The patient, a 69-year-old male, had a 20-year history of splenectomy after splenic and diaphragmatic injury following a severe motor vehicle accident. His medical background was previous cigarette smoking with a 60 pack-year history, and he has been smoke-free for 1 year. He presented with thoracic pain for 1 month, without other respiratory symptoms. A chest computed tomography (CT) plain scan showed a pulmonary mass in the left lower lobe on December 14, 2023. On December 19, 2023, the patient had an enhanced chest CT that showed “the left lower lobe pulmonary mass was approximately 3.3 cm × 2.3 cm in size, not clearly demarcated from the adjacent diaphragm, with slightly lobed edges and contrast-enhanced masses, enlargement of hilar or mediastinal lymph nodes” (Fig. 1). On the same day, lung cancer-related tumor markers including carcinoembryonic antigen, squamous cell carcinoma-related antigen, and gastrin-releasing peptide precursor were normal. On December 19, 2023, he underwent a CT-guided percutaneous needle biopsy along the pleura, which biopsy specimen was ultimately confirmed splenic tissue (Fig. 2). Immunohistochemical results: CK(-), EMA(-), Vimentin(+), TTF-1(-), Napsin A(-). Further investigation of the cause of chest pain, coronary angiography examination confirmed as severe stenosis of the main coronary artery and 3 branches of the coronary. To date, follow-up assessments indicate that pulmonary mass was unchanged in appearance compared to his prior imaging. The patient has given informed consent for the publication of this case report.

## 3. Discussion

Thoracic splenosis, first reported by Shaw and Shafi in 1937, involves the ectopic transplantation of splenic tissue into the thoracic cavity after splenic injury or resection. It is a rare phenomenon, with approximately 18% of cases associated with splenic rupture.^[3]^ It is most commonly observed in patients with a history of severe splenic trauma or surgery, primarily due to gunshot wounds or road traffic accidents, accounting for 36% and 50% of reported thoracic splenosis cases, respectively.^[3]^ Proposed mechanisms^[4]^ include traumatic splenic rupture leading to migration of splenic fragments through diaphragmatic defects into the thoracic cavity, where they implant and grow as ectopic splenic tissue. Hematogenous spread of splenic pulp and hypoxia in duced tissue growth have been suggested as well. Recent literature on thoracic splenosis mainly consists of case reports, with <100 cases reported in the literature thus far. The average interval between the causal event and detection of thoracic splenosis is 21 years (range 3–45 years).^[5]^

Most cases are incidentally detected on imaging, with no specific symptoms. Chest CT often reveals pleural or pulmonary parenchymal nodules, occasionally with calcifications (reported in a minority of cases).^[6]^ Differentiation from malignancies, such as mesothelioma or metastatic tumors, is critical due to the high misdiagnosis rate.^[1,2]^ Technetium-99m (Tc-99m) heat-damaged erythrocyte nuclear imaging, together with detailed history, is the gold standard for diagnosis.^[7]^ The thoracic functional spleen may increase in size over time, potentially mimicking malignancy. Histopathological biopsy demonstrates characteristic red pulp and white pulp structures of splenic tissue without evidence of malignancy.^[4]^ The role of PET scan in identifying splenic tissue is limited, as it is not specific.^[8]^

Asymptomatic patients typically require no intervention and are monitored with regular follow-up. Surgery is indicated only for complications (e.g., pleural effusion, hemoptysis) or when malignancy cannot be ruled out. A critical complication of splenectomy is overwhelming post-splenectomy infection, which can occur up to 40 years after surgery. Notably, implanted splenic tissue may partially compensate for the asplenic state.^[9]^

The patient presented with only thoracic pain which was unrelated to the mass found during admission. Detailed review of history revealed that he had a 20-year history of splenectomy after splenic and diaphragmatic injury following a severe motor vehicle accident. CT-guided percutaneous needle biopsy specimen was ultimately confirmed splenic tissue. As demonstrated in most cases, thoracic splenosis was not clearly demarcated from the adjacent diaphragm, pericardium and thoracic wall. Like most patients diagnosed with thoracic splenosis, the patient did not require surgical intervention. And we believe that percutaneous needle biopsy may be avoided with detailed history of trauma and splenectomy, and Tc-99m heat-damaged erythrocyte nuclear imaging.

Thoracic splenosis is a rare complication of splenic rupture, diagnosed based on trauma history, imaging, and nuclear medicine studies. While most patients remain asymptomatic, the risk of misdiagnosis as malignancy necessitates caution. Conservative observation is the mainstay of management, with surgical intervention reserved for specific indications. Given the rarity of this condition, establishing multicenter case registries and databases will advance research and improve clinical practice.

## Author contributions

**Data curation:** Baoxiang Pei, Fen Pan.

**Formal analysis:** Baoxiang Pei, Fen Pan.

**Project administration:** Baoxiang Pei, Fen Pan.

**Supervision:** Zhiliang Hu.

**Validation:** Zhiliang Hu.

**Visualization:** Zhiliang Hu.

**Writing – original draft:** Baoxiang Pei, Fen Pan.

**Writing – review & editing:** Baoxiang Pei, Zhiliang Hu, Fen Pan.
